# Degradation of 4-Chlorophenol in Aqueous Solution by Sono-Electro-Fenton Process

**DOI:** 10.20964/2018.09.46

**Published:** 2018-08-05

**Authors:** Roya Nazari, Ljiljana Rajić, Yunfei Xue, Wei Zhou, Akram N. Alshawabkeh

**Affiliations:** 1 Department of Civil and Environmental Engineering, Northeastern University, 400 Snell Engineering, 360 Huntington Avenue, Boston, MA 02115, USA.; 2 Department of Energy Science and Engineering, Harbin Institute of Technology, Harbin 150001, P.R. China; 3 State Environmental Protection Key Laboratory of Environmental Risk Assessment and Control on Chemical Process, East China University of Science and Technology, Shanghai 200237, P.R. China

**Keywords:** Electro-Fenton process, Sono-Electro-Fenton process, 4-chlorophenol, Advanced oxidation processes, Pd catalysts

## Abstract

Electro-Fenton (EF) and ultrasound radiation (US) have been of interest for the removal of chlorinated compounds from water. This study evaluates the effects of different parameters on sono-electro-Fenton (SEF) for degradation of 4-chlorophenol (4-CP) in an aqueous solution. This study uses pulsing US waves along with Pd-catalyzed EF to degrade contaminants in water while maintaining temperature. The usage of pulsing US waves along with Pd catalyzed EF to remove contaminants while maintaining temperature has not been reported previously. SEF ability to degrade 4-CP was compared with the performance of each process (EF and sonolysis) alone. Initial pH, current density, background electrolyte, Fe^2+^ concentration, Pd/Al_2_O_3_ catalyst concentration, US waves, and sonifier amplitude were optimized in a two electrode (Ti/mixed metal oxide or Ti/MMO) batch system. The degradation of 4-CP increased from 1.85% by US to 83% by EF to nearly >99.9% by coupled SEF. With US radiation under 70% amplitude and 1:10 ON/OFF ratio, the removal rate of 4-CP increased to 98% compared to 62% under EF alone within the first 120 min in the presence of 80 mg L^−1^ Fe^2+^, 16.94 mA cm^−2^ of current density, 1 g L^−1^ Pd/Al_2_O_3_ catalyst (10 mg Pd), and initial pH of 3. However, the degradation rate decreased after 120 min of treatment, and complete 4-CP removal was observed after 300 minutes. The sonolysis impacted the 4-CP removal under coupled SEF, mostly due to the contribution of mass transfer (micromixing), while radical formation was found to be absent under the conditions tested (20kHz). The pulsed US was found to increase the temperature by only 8.7°C, which was found not to impact the 4-CP volatilization or degradation. These results imply that low-level US frequency through pulses is a practical and efficient approach to support electro-Fenton reaction, improving reaction rates without the need for electrolyte cooling.

## 1. INTRODUCTION

Degradation of organic and inorganic impurities in fresh water, drinking water, wastewater, and groundwater occurs through different electrochemical treatments including electrochemical flotation, electrochemical coagulation, electrochemical reduction, electrodeposition and electro-oxidation. To treat wastewater, disinfect drinking water, or enhance the remediation of polluted soils, electrochemical techniques have been applied extensively, especially electro-oxidation treatment [1–3].

Chlorophenols (CPs) contain a group of environmental contaminants that are identified as priority toxic pollutants by the US Environmental Protection Agency (EPA) [4,5] and the European Union (EU) [6], which identified 0.5 mg L-1 as the upper permissible limit in publicly supplied water [7]. 4-CP is a toxic, persistent substance that was widely used in herbicides, pesticides, and disinfectants.

Various treatment methods have been investigated for transformation of 4-CP into less toxic forms [1]. Degradation of CP has been evaluated using oxidation processes such as electrochemical incineration [2], anodic oxidation [3,8], photolytic oxidation [9], biological processes [10], ultrasound (US) [11–13], electro-Fenton (EF) [3], and peroxi-coagulation processes [3]. Electrochemical treatments are recognized to be environmentally-friendly as they can be performed in situ without external chemical additions [14–16], which lowers the cost of remediation. Contaminants can be electrochemically transformed via direct or indirect oxidation and/or reduction mechanisms [1,17,18].

The Fenton reaction was shown to be effective for the transformation of chlorinated solvents in groundwater [19]. The Fenton reaction is based on the transformation of hydrogen peroxide (H_2_O_2_) in the presence of Fe^2+^ into highly reactive Hydroxyl radicals (^•^OH) (Eq. 1). Fe^2+^ then regenerates via reduction with H_2_O_2_ (Eq. 2).

Eq. 1 $${\text{Fe}}^{\text{2+}}+\;{\text{H}}_{\text{2}}{\text{O}}_{\text{2}}\to {\text{Fe}}^{\text{3+}}+{\;}^{•}\text{OH+}{\text{OH}}^{-}$$

Eq. 2 $${\text{Fe}}^{\text{3+}}+\;{\text{H}}_{\text{2}}{\text{O}}_{\text{2}}\to {\text{Fe}}^{\text{2+}}+\;{\text{HOO}}^{•}{\text{+H}}^{+}$$

The Fenton reaction can also be initiated, supported, and maintained by electrochemical processes. In the Pd-catalyzed EF reaction, H_2_O_2_ is produced in situ through the reaction between electro-generated O_2_ and H_2_ (Eq. 3), and further activated to ^•^OH (Eq. 4) [20–23]. The EF method allows better control of H_2_O_2_ and ^•^OH generation, accelerates the production rate of ^•^OH compared to traditional Fenton’s method, and supports reduction of Fe^3+^ to Fe^2+^ at the cathode (Eq. 5) [1]. Although an in situ production of H2O2 can be hindered by the low solubility of oxygen in water [20,23,24], a Pd catalyst has been proven to enhance its production rate.

Eq. 3 $${\text{H}}_{\text{2}}{\text{+O}}_{\text{2}}\to {\text{H}}_{2}{\text{O}}_{2}$$

Eq. 4 $${\text{H}}_{2}{\text{O}}_{\text{2}}+{\text{Fe}}^{\text{2+}}{\text{+H}}^{+}\to {\text{Fe}}^{3+}+{\;}^{•}{\text{OH}+\text{H}}_{2}\text{O}$$

Eq. 5 $${\text{Fe}}^{3+}{\text{+e}}^{-}\to {\text{Fe}}^{\text{2+}}$$

Ultrasonic radiation has also demonstrated a great potential for various applications, including the intensification of chemical synthesis, cleaning, and water treatment [9–11,25]. Application of frequencies above 20 kHz causes growth of cavitation bubbles which become unstable after a number of cycles. Upon collapsing, each of the bubbles acts as a hotspot, generating energy to increase the temperature (up to 5,000 K) and pressure (up to 500 atm) with cooling rates as fast as 109 K/s. There are many parameters affecting the cavitation and bubble collapse process (sound wave frequency and intensity, external pressure and temperature, solvent characteristics, and presence of soluble gases), and, consequently, the impact of sonolysis on the degradation of the contaminants in aqueous solutions [8,13,26].

Sonolysis occurs through three reaction zones; the gas phase region inside the bubble (pyrolysis reactions), the interfacial region (reactions occurring in pressure/temperature gradients in aqueous phase), and the bulk solution [13,26,27]. The chemical effect of the applied acoustic field is the sonolysis of water molecules and thermal dissociation of oxygen molecules (when present), which produce different kinds of reactive species (^•^OH, H^•^, O^•^ and hydroperoxyl radicals (OOH^•^) (example given in Eq. 6 where the sign ‘)))’ denotes US power).

Eq. 6 $${\text{H}}_{\text{2}}\text{O}+)))\to {\;}^{•}\text{OH}+{\text{H}}^{•}$$

The physical effects of US irradiation are induced by cavitation bubbles (micro-jets and shockwaves) and by propagation of US waves through a liquid medium (streaming) [13]. Where surface instabilities occur, cavity collapse is very asymmetric and generates high-speed jets (micro- jets) of liquid causing the enhancement of the rate of mass transfer [28–30].

An application of an ultrasonic field along with electrochemical processes suppresses gas bubble accumulation on the surface of electrode. Acoustic waves will oscillate the adhered gas bubbles at the surface of electrode leading to removal of gas bubbles from the surface of electrode through the waves’ vibration [29,31]. Several studies have combined US radiation with other advanced oxidation processes (AOPs), including using EF to remove chlorinated compounds from aqueous solutions [7,32–37]. These coupled processes have resulted in an increase of contaminant removal in comparison with using each method separately.

Trabelsi et al. (1996) used 500KHz US radiation along with EF (current density=68 Am^−2^) and showed that a total degradation of phenol within 20 minutes with no production of toxic intermediates is possible [38]. Yasman et al. used US radiation (20 KHz) along with EF mechanism to treat 2,4- dichlorophenxyacetic acid (2,4-D) and its derivative 2,4-dichlorophenol (2,4-DCP). They accomplished almost 50% oxidation of 2,4-D solution (300 ppm) in only 60 seconds, while complete removal was achieved after 10 minutes [39]. Liang et al. investigated the impact of US on a Fenton- like reaction for 4-chlorophenol removal from water, and found that under tested conditions, 4-CP was completely decomposed within 2 min of ultrasonic irradiation when its initial concentration in the solution was 100 mg/l [28]. In another study, Mehmet et al. used an undivided electrolytic cell with a Pt anode and a 3-dimensional carbon-felt cathode to carry out EF and SEF oxidation for three contaminants, 2,4-dichlorophenoxyacetic acid (2,4-D), 4,6-dinitro-o-cresol (DNOC), and the synthetic azo dye azobenzene (AB). It was observed that synergistic effect between EF and US provides a higher degradation rate than that provided by the two techniques separately for 2,4-D and DNOC [6].

The objective of this study is to investigate oxidation of 4-CP by Pd-catalyzed EF process coupled with sonolysis using pulsed US waves. While the benefits of US pulse by boron-doped diamond electrodes were reported [40], the application of pulsing US waves along with Pd-catalyzed EF reaction has not been reported. In this research, we examine the application of US waves at ON/OFF ratio of 0.1 (US was ON: 5.9 sec. and OFF: 59 sec). The performance of EF under different initial pH, Fe^2+^ concentration, palladium (Pd) catalyst concentration, background electrolytes, and current densities was also tested. We also evaluated the simultaneous use of Fe anode in different application regimes as a practical, in situ supply of ferrous iron. The SEF tests were conducted under optimum conditions, contaminant removal by SEF process was compared with both EF and sonolysis, and the mechanisms involved were then evaluated.

## 2. MATERIALS AND METHODS

### 2.1 Materials

4-chlorophenol (C_6_H_5_ClO, 99+ %) and palladium catalyst (Pd/Al_2_O_3_, 1% Pd on alumina powder, with a specific surface area of 150 m^2^g^−1^) were purchased from Acros and Alfa Aesar, respectively. Phenol (C_6_H_5_OH, 89.6%), sodium sulfate anhydrous (Na_2_SO_4_, 99%), sulfuric acid (H_2_SO_4_, 98%), sodium bicarbonate (NaHCO_3_, 99–100%), acetonitrile (99.8+ %), sodium hydroxide (NaOH, 96%), benzoic acid (C_7_H_6_O_2_, 99.9+%), 4-hydroxylbenzoic acid (C_7_H_6_O3, 99.9+%), o- Phosphoric acid (H_3_PO_4_, 85%), and Acetic Acid (Glacial, HPLC grade) were acquired from Fisher Scientific. Ferrous sulfate (FeSO_4_.7H_2_O, pro analysis) was obtained from J.T. Baker Analyzed. Palladium catalyst (1% on carbon 4 to 8 mesh), Potassium-hydrogen phthalate (C_8_H_5_KO_4_), HPLC grade water, and methanol were bought from Sigma-Aldrich. The syringe filters with 0.22 μm and 0.45 μm pore sizes were purchased from Millex. Titanium sulfate (TiSO_4_, 65%) was obtained from GFS Chemicals. All solutions were prepared in de-ionized water (18.2 mΩ.cm), obtained from a Millipore Milli-Q system.

### 2.2. Experimental setup

**Figure 1.**
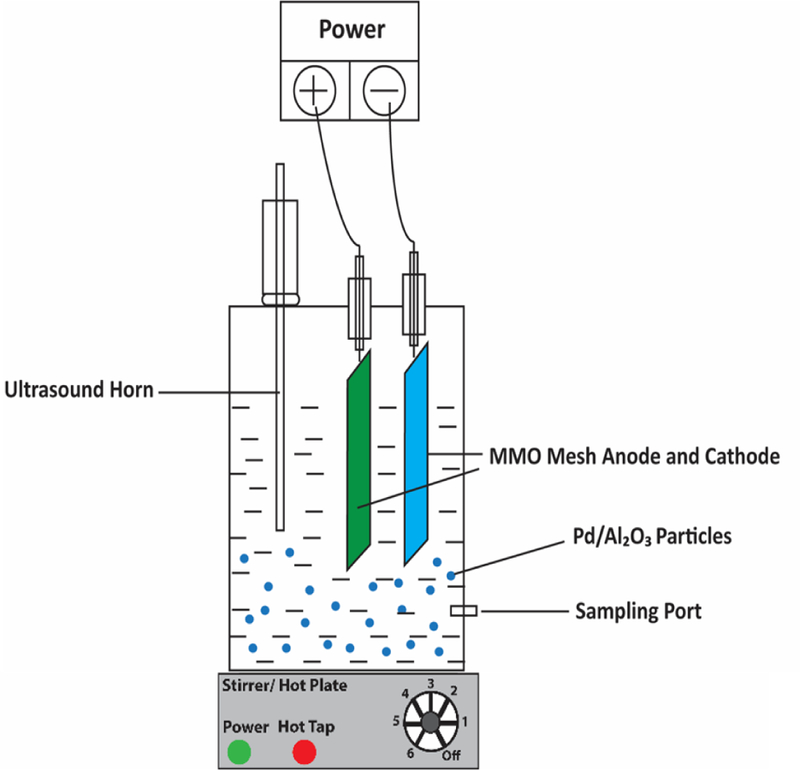
Batch setup

As shown in Figure 1, a one-liter acrylic cell with an 11.4 cm inner diameter and a 10 cm height was used as batch reactor. Two Ti-based mixed metal oxide meshes (Ti/MMO, IrO_2_/Ta_2_O_5_ coating on titanium mesh type, 3N international, USA) with 3.6 cm in diameter, 1.8 mm thickness, and a surface area of 11.8 cm^2^ were used as both anode and cathode. The distance between the electrodes was 6 cm. The synthetic contaminated groundwater was prepared by adding 4-CP to achieve a concentration of 200 ppm in the electrolyte (10 mM Na_2_SO_4_, NaHCO_3_ or NaNO_3_) with different Fe^2+^ concentrations. An alternative to the addition of ferrous sulfate as the Fe^2+^ source was using a cast iron anode with dimensions of 85×15×1.8 mm (length × width × thickness) to produce Fe^2+^ in-situ. The current was split between Ti/MMO and iron anodes by a rheostat, where the current applied to the iron anode was calculated to supply a total of 80 ppm Fe^2+^ (based on Faraday’s law assuming the charge transfer between electrode surface and electrolyte is a 100% faradaic process). Fe^2+^ production was tested for optimum EF in the following conditions: during the entire 240 min by applying 2.85 mA cm^−2^ to iron anode (with a surface area of 6.3 cm^2^), during the first 30 minutes of treatment under 22.85 mA cm^−2^, and following a 30-minute delay of treatment under 3.17 mA cm^−2^ supplied to the iron anode. The iron anode’s ON/OFF periods were applied based on the estimated H_2_O_2_ production, which reaches maximum values after 30 min.

The use of an iron anode in the electrochemical cell allowed for the generation of a wide range of Fe^2+^ concentrations without relying solely on the naturally present amounts of Fe^2+^ in the groundwater (concentration rarely exceeds 50 ppm). Sulfuric acid and sodium hydroxide were used to adjust the pH of the electrolyte. After adding the synthetic groundwater, defined Fe^2+^, and Pd/Al_2_O_3_ catalyst concentrations, the cell was sealed and the solution was stirred at a rate of 180 rpm using a magnetic stirrer. As summarized in Table 1, the influence of different parameters on EF towards 4-CP transformation were tested.

During SEF and sonolysis tests, a sonifier (20 KHz Branson Ultrasonics Co.) with a 7.7 cm titanium horn was placed in the reactor and defined US amplitudes were applied in pulses. The SEF tests were conducted under 80 mg L^−1^ Fe^2+^, 10 mg L^−1^ Pd/Al_2_O_3_ powder, initial pH of 3 and 16.94 mA cm^−2^ current density, with the amplitudes (%) of: 10, 30, 50, and 70 using ON/OFF pulse ratio of 0.1 and 10% to 30% with ON/OFF=0.2.

### 2.3. Analysis

At specific times, 2 ml of solution were collected from the sampling port (located 2.4 cm from the bottom of the reactor, Figure 1) and were filtered through a 0.22 μm pore size syringe filter. 4-CP and phenol concentrations were measured by a 1200 Infinity Series HPLC (Agilent) equipped with a 1260 DAD detector and a Thermo ODS Hypersil C18 column (4.6 × 50 mm) with a 5 µm particle size. Mobile phase was a mixture of methanol, water, and glacial acetic acid (49:49:2) with a 1 mL min^−1^ flow rate. Detection wavelength was 254 nm. The retention time was 2.5 min for phenol and 4.34 min for 4-CP [41]. Total organic carbon (TOC) measurements were performed by a TOC analyzer, TOC-L CPH-CPN (Shimadzu, Japan), after sample filtration through 0.45 μm pore size filters (Millipore), and acidification (pH≤2) with concentrated HCl. The 4-CP removal % was calculated by (Eq. 7):

Eq. 7 $$\text{Removal}\left(\%\right)=\;\frac{{\text{C}}_{0}-{\text{C}}_{\text{t}}}{{\text{C}}_{0}}*100,$$

where C_0_ is the initial concentration of 4-CP (mg L^−1^) and C_t_ is 4-CP concentration at time t during treatment (mg L^−1^). pH and dissolved oxygen (DO) were measured by pH meter and DO meter (Thermo Scientific). H_2_O_2_ was measured at 405 nm on a Shimazu UV-Vis spectrometer after coloration with TiSO_4_. Benzoic acid (BA) was used as hydroxyl radical probe [4] and was measured along with p-hydroxybenzoic acid (pHBA) by a 1200 Infinity Series HPLC (Agilent) equipped with a 1260 DAD detector (Agilent) and a Thermo ODS Hypersil C18 column (4.6 × 50 mm) with a 5 µm particle size. The initial BA concentration was 5 mM. 1 mL samples were taken at the predetermined time intervals and samples were then filtered by the 0.22μm filter (PVDF). The treated samples were then quenched by 0.1 mL ethanol before the HPLC analysis. The mobile phase was a mixture of methanol and 0.1% phosphoric acid (20:80) with a 0.5 mL min^−1^ flow rate. The detection wavelengths for BA and p-HBA were 280 nm and 210 nm, respectively. The retention times for BA and p-HBA were 14.0 min and 5.0 min, respectively. Both BA and p-HBA were stable throughout the analytical process.

## 3. RESULTS AND DISCUSSION

### 3.1 Electro-Fenton (EF) Optimization

#### 3.1.1 Influence of different Fe^2+^ concentrations

**Figure 2.**
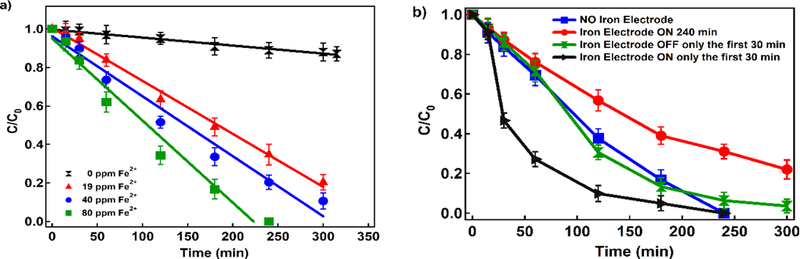
a) Effect of Fe^2+^ concentration on 4-CP decay, and b) effect of iron anode on 4-CP decay (Conditions: different Fe^2+^ concentrations, current density: 16.94 mA cm^−2^, 10 mg L^−1^ Pd as Pd/Al_2_O_3_, Na_2_SO_4_:10 mM, pH=3, and 4-CP: 200 ppm)

The impact of initial Fe^2+^ concentration on 4-CP removal was examined under concentrations of 20, 40 and 80 mg L^−1^, and the 4-CP decay over time is presented in Figure 2a. Preliminary tests showed that pH=3 provides a higher removal percentage, which is in accordance with other studies [23,42]. The degradation of 4-CP via EF reaction follows zero-order kinetics, indicating that degradation is limited by the availability of the reactive ^•^OH (Table 2). The 4-CP degradation rate increased from 0.0004 min^−1^ in the absence of Fe^2+^ to 0.0043 min^−1^ in the presence of 80 mg L^−1^ Fe^2+^. In the absence of Fe^2+^, only 11% of 4-CP was removed via (i) indirect hydrodechlorination at the Ti/MMO cathode, and/or (ii) Pd-catalyzed reduction processes [23]. The results show that an increase in Fe^2+^ increases 4-CP degradation as it increases ^•^OH concentration reactions.

In order to evaluate the mechanism of 4-CP removal, EF was performed in the presence of two different concentrations of tert-butyl (^•^OH scavenger) (Eq. 8) [1]. A significant change in 4-CP removal was observed after 60 minutes of treatment in the absence and presence of the radical scavenger; the 4-CP zero-order decay rate in the absence and presence of tert-butyl are 0.0041 min^−1^ and 0.0016 min^−1^, respectively. Also, changes in degradation rate during 60 minutes of treatment in the presence of tert-butyl were negligible. This indicates that ^•^OH are the primary reactive species responsible for 4-CP degradation via electro-Fenton reaction supported by Pd catalyst under conditions tested (approx. 75%) [1].

Eq. 8 $${\left({\text{CH}}_{\text{3}}\right)}_{3}\text{COH}+{\;}^{•}\text{OH}\to {\left({\text{CH}}_{3}\right)}_{2}{\;}^{•}{\text{CH}}_{\text{2}}{\text{COH}+\text{H}}_{2}\text{O}$$

In order to continuously supply the system Fe^2+^ and delay the redox of ferrous ion to ferric ion, an iron electrode was used instead of externally adding Fe^2+^ [31,43]. In addition, the capability to maintain *in situ* Fe^2+^ sources are valuable for the treatment of groundwater with low natural Fe^2+^ content. Iron anodes have multiple advantages: (i) Fe^2+^ is continuously released from the sacrificial iron anode, (ii) manipulating the current density controls the electrolytic production of Fe^2+^, (iii) by reversing the polarity of iron electrode, the generation of Fe^2+^ can be prevented or suppressed [31,44,45], and iron anode reactions demand less energy [46]. Figure 2b shows the comparison of the system’s performance for 4-CP removal in the presence of iron anode and external Fe^2+^ addition. The EF system is most effective (>99.9% removal) when the iron anode was operating for the first 30 min of testing because Fe^2+^ is continuously produced *in situ* by anodic corrosion.

#### 3.1.2 Pd catalyst

**Figure 3.**
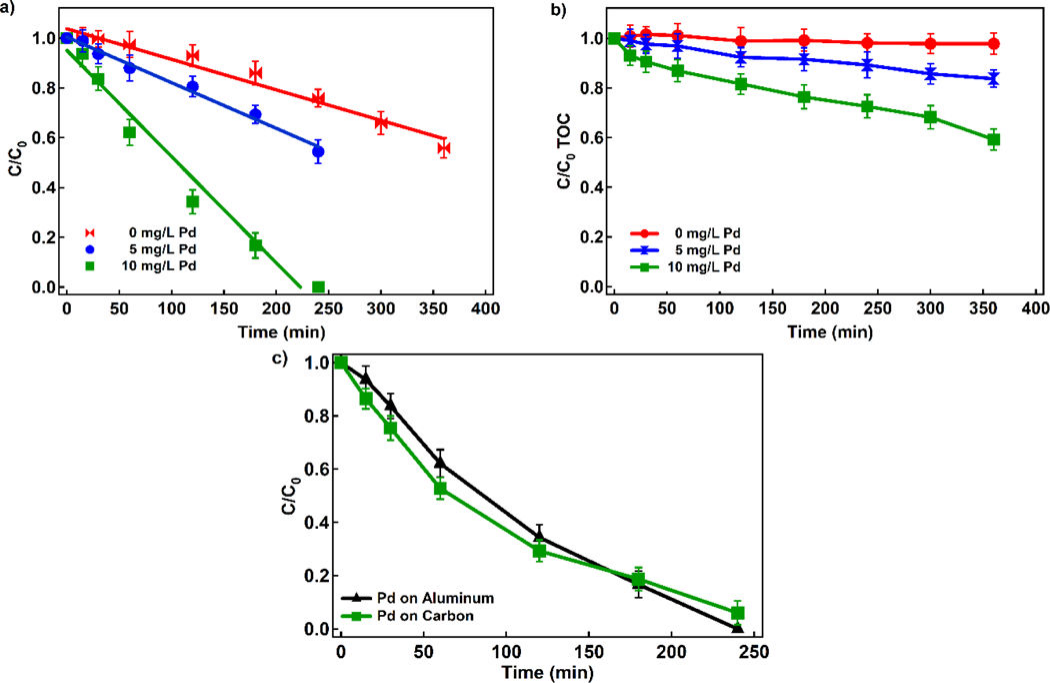
a) Degradation profiles of 4-CP using different Pd/Al_2_O_3_ concentrations, b) degradation profiles of 4-CP using different Pd/Al_2_O_3_ concentrations on TOC, and c) degradation profiles of 4-CP using different types of Pd (Conditions: Fe^2+^: 80 ppm, current density: 16.94 mA cm^−2^, different Pd/Al_2_O_3_ concentrations, Na_2_SO_4_:10 mM, pH=3, and 4-CP: 200 ppm)

The amount and type of Pd catalyst influences the rate of H_2_O_2_ production. Figure 3a shows 4-CP concentration profile over time in the presence of different Pd/Al_2_O_3_ concentrations. Under similar conditions, an increase in Pd/Al_2_O_3_ concentration from 0 to 10 mg L^−1^ increased 4-CP removal from 24% in 5 hours to >99.9% in less than 4 hours. The Zero-order rate constant for 4-CP removal increased from 0.0012 min^−1^ in the absence of Pd/Al_2_O_3_ to 0.0043 min^−1^ in the presence of 10 mg L^−1^ Pd. Decay of 4-CP in the absence of Pd indicates that processes on Ti/MMO electrodes contribute to 4-CP degradation but the rate and overall removal is low; the H_2_O_2_ electrogeneration can occur via two electron oxygen reduction, but the amount is approximately 30% of the amount produced in Pd presence. The addition of a catalyst significantly increased removal due to the production of higher H_2_O_2_ concentrations and, consequently, production of ^•^OH. The correlation between Pd/Al_2_O_3_ concentration and H_2_O_2_ production has previously been proven [23].

The influence of catalyst concentration was also evaluated based on TOC removal in addition to 4-CP transformation and decay (Figure 3b). The decay in TOC during the treatment indicates the total mineralization of the parent compound (4-CP) and its oxidation byproducts. Similar to 4-CP decay, higher Pd concentrations increase TOC removal rates. However, the overall TOC removal is limited (% with 10 mg Pd L^−1^) indicating that 4-CP transforms into other dissolved organic compounds (e.g., phenol) within the first six hours of treatment. Prolonging the treatment to 10 hours removed up to 85% of TOC since ^•^OH are continuously generated during EF, causing total mineralization of 4-CP and its byproducts.

In addition to Pd concentration, we also tested the influence of Pd support type on the overall degradation efficiency. Although Pd/Al_2_O_3_ is a commercial catalyst extensively investigated for catalytic oxidation of volatile organic carbons (VOCs), we tested Pd on active carbon (Pd/C) as alternative catalyst support. While both catalysts support the 4-CP removal (Figure 3c), the transformation pathways significantly differ. Based on the control experiments (without current), 59.71% of 4-CP was removed from the solution in 240 min when Pd/C was applied while 4-CP concentration decay was only 3.23% when Pd/Al_2_O_3_ was used.

Absorption rate of Pd/AL_2_O_3_ is lower than Pd/C, which is consistent with other studies [42]. This indicates that Pd/C supports 4-CP sorption over EF reaction and, although removal rate and efficiency is significant, Pd/C is not suitable catalyst for 4-CP degradation via EF reaction.

Because of the intrinsic properties of Al, such as its low standard reduction potential, high abundance, high reactivity, stability, and inexpensiveness, Pd/Al_2_O_3_ was used as a catalyst type in all experiments.

#### 3.1.3 Current density

**Figure 4.**
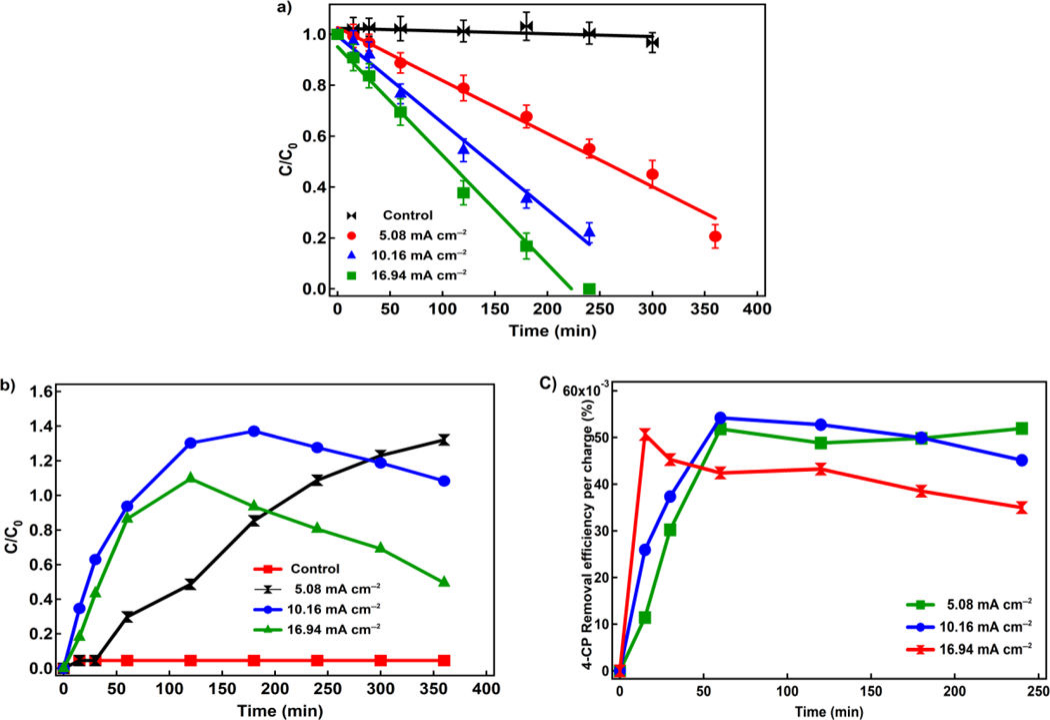
a) Degradation profile of 4-CP in different current densities (Conditions: Fe^2+^: 80 ppm, Pd/Al_2_O_3_: 10 mg L^−1^, Na_2_SO_4_: 10 mM, pH=3 and 4-CP: 200 ppm); b) Degradation profile of phenol in different current densities; and, c) 4-CP removal efficiency per charge (Conditions: Fe^2+^: 80 ppm, 10 mg L^−1^ Pd as Pd/Al_2_O_3_, Na2SO4:10 mM, pH=3, and 4-CP: 200 ppm)

Electric current is a crucial factor affecting the EF process as it is directly related to the formation of H_2_O_2_, the regeneration rate of Fe^2+^, and consequently the generation rate of ^•^OH. To investigate the effect of applied current density on the oxidation of 4-CP, several experiments were performed with different current densities in the range of 0 to 16.94 mA cm^−2^ at the optimum Fe^2+^ concentration of 80 mM (Figure 4a). The decay rate increases from 0.0001 min-1 to 0.0043 min^−1^ when current density increases from 0 mA cm^−2^ to 16.94 mA cm^−2^. Increasing the current density accelerates the 4-CP decay due to progressively larger generation of H_2_O_2_ as water electrolysis to oxygen and hydrogen [20,23,47].

Although current efficiency (calculated based on Faraday’s law) indicates that utilization of 16.94 mA cm^−2^ is significantly less compared to 10.16 mA cm^−2^, we conducted all our tests under 16.94 mA cm^−2^. This is based on the effects of current density on the degradation profile of phenol, a 4-CP degradation byproduct that was analyzed during the treatment (Figure 4b). Since the main focus of this study was to evaluate the feasibility of using US to enhance electro-Fenton reaction, further analysis is needed to identify all oxidation byproducts.

Under 16.94 mA cm^−2^, phenol concentration significantly decays after 120 minutes of treatment, while under 10.16 mA cm^−2^ similar behavior occurs after 180 minutes. Under 5.08 mA cm^−2^, phenol concentration continues to increase, indicating that ^•^OH was mainly utilized for 4-CP oxidation. The results indicate that under 16.94 mA cm^−2^, ^•^OH was partially utilized for phenol oxidation in the first 200 minutes of treatment, and completely utilized for phenol and other byproducts after 250 minutes since complete degradation of 4-CP is achieved.

Charge transferred between electrode and electrolyte was calculated. Then, at each time point, removal efficiency was divided by the charge to produce Figure 4c. Figure 4c shows removal efficiency of 4-CP per charge versus time, which are 45%, 46%, and 38% for 5.08 mA cm^−2^, 10.16 mA cm^−2^ and 16.94 mA cm^−2^ respectively. As can be seen in the figure, i=16.94 mA cm^−2^ has lower removal per charge transferred, meaning that at i=16.94 mA cm^−2^ some charges transferred are not being used in the 4-CP oxidation reactions.

#### 3.1.4 Background electrolyte

**Figure 5.**
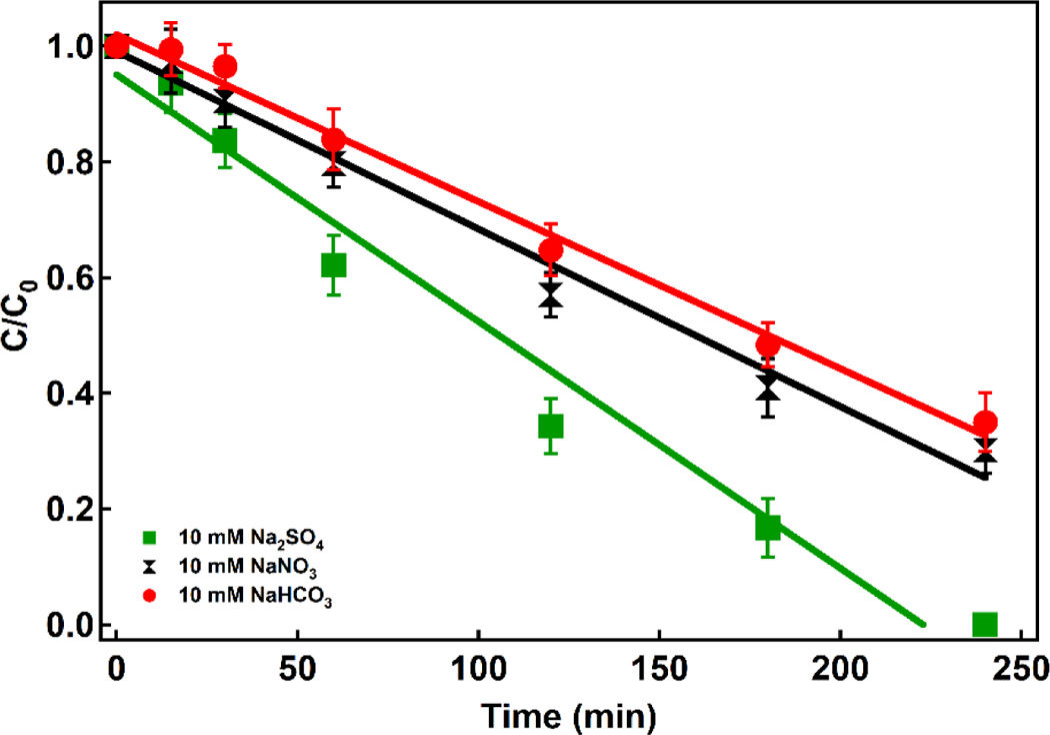
Degradation profile of 4-CP in different background electrolytes (Conditions: Fe^2+^: 80 ppm, current density: 16.94 mA cm^−2^, 10 mg L^−1^ Pd as Pd/Al_2_O_3_, pH=3, and 4-CP: 200 ppm)

Background electrolytes affect EF performance because they improve the solution conductivity and can either support or hinder the efficiency of EF reactions. Figure 5 shows the concentration of 4-CP over time in the presence of 10 mM NaNO_3_, 10 mM Na_2_SO_4_ and 10 mM NaHCO_3_. The least 4-CP removal appears in the presence of 10 mM NaHCO_3_ (k=0.0029 min^−1^) while the most removal is the system containing 10 mM Na_2_SO_4_ (k=0.0043 min^−1^). The possible effects of inorganic anions on the electro-Fenton reaction include: (i) effect of ionic strength, (ii) complexation or precipitation of iron species, (iii) scavenging of ^•^OH and formation of less reactive inorganic radicals, and (iv) oxidation including these inorganic radicals [48].

In EF systems, NaHCO_3_ suppresses performance since it is not a strong electrolyte but, more importantly, because HCO_3_^−^ acts as a ^•^OH scavenger [29,49]. Although scavenging leads to formation of carbonate radicals, the reduction potential is less (E=1.5V) than that of ^•^OH (E=2.43 V), meaning that the general oxidative activity in the system depletes. Further, in the pH range used in the study, a Fe(CO_3_) complex is expected to be the most kinetically active Fe^2+^ species for the H_2_O_2_ activation [50].

### 3.2. Sono-electro-Fenton (SEF)

#### 3.2.1. Optimization of ultrasound (US) amplitude

**Figure 6.**
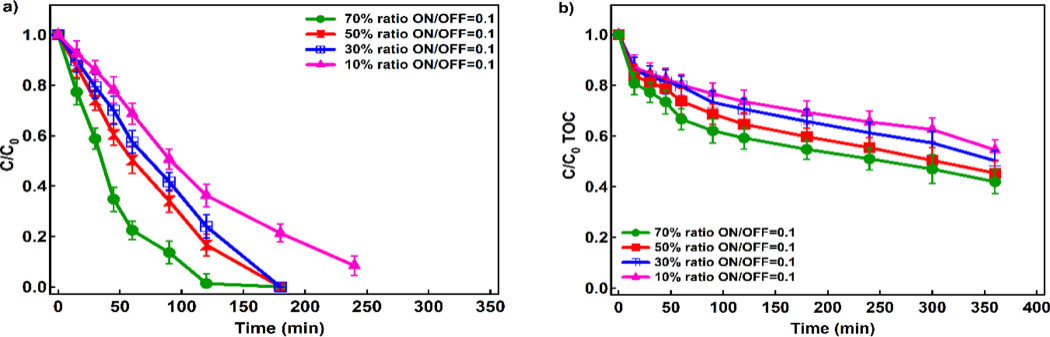
a) Degradation profile of 4-CP over time with different amplitudes and ON/OFF ratios, b) TOC over time with different amplitudes and ON/OFF ratios (Conditions: Fe^2+^: 80 ppm, current density: 16.94 mA cm^−2^, 10 mg L^−1^ Pd as Pd/Al_2_O_3_, Na_2_SO_4_: 10 mM, pH= 3, and 4-CP: 200 ppm)

We applied sonification in pulses to avoid the need to cool the system thus increasing the practicality of the US use [40]. Optimum EF parameters (200 mg L^−1^ 4-CP as an initial concentration, 80 mg L^−1^ Fe^2+^, 16.94 mA cm^−2^ of current density, 10 mg L^−1^ Pd/Al_2_O_3_ catalyst and initial pH of 3) were used to identify the best sonifier amplitude. Figure 6a shows 4-CP concentration decay over time in the presence of different US amplitudes. Under identical ON/OFF ratios of 0.1, an increase in amplitude increased 4-CP removal; for example, amplitude of 70% increases the 4-CP removal 1.5 times in comparison with an amplitude of 10%. In addition, TOC over time in the presence of different US amplitudes shows that larger amplitudes result in up to 59% TOC removal (Figure 6b), indicating more sufficient mineralization compared to other amplitudes.

#### 3.2.2. Ultrasound (US) impact on 4-CP removal

**Figure 7.**
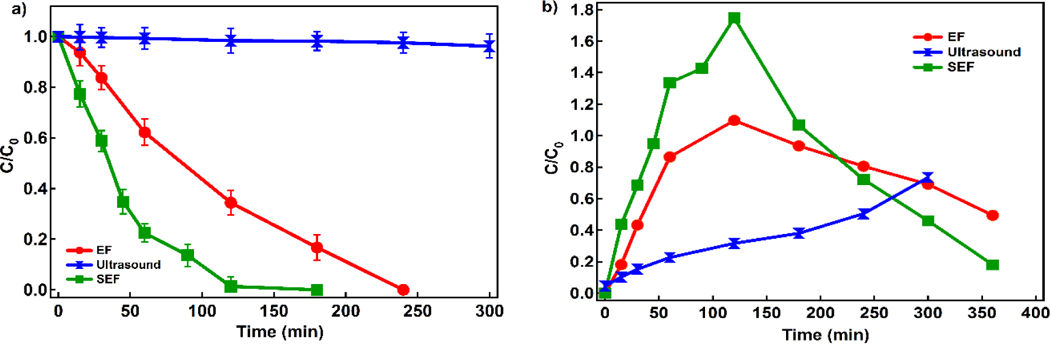
a) 4-CP and b) phenol decay during EF, US and SEF (Conditions: Fe^2+^: 80 ppm, current density: 16.94 mA cm^−2^, 10 mg L^−1^ Pd as Pd/Al_2_O_3_, Na_2_SO_4_: 10 mM, pH= 3, and 4-CP: 200 ppm)

Comparison of performance under EF, US and SEF. In order to evaluate the impact of US on 4-CP degradation, we conducted sonolysis of 4-CP performed under optimum amplitude (70%) and ON/OFF ratio of 0.1. As presented in Figure 7a, application of US alone removed only 1.85% of 4-CP while SEF and EF achieved removal of >99.9% and 83%, respectively. Besides 4-CP decay, SEF shows favorable phenol decay profile (Figure 7b). Garbellini et al. found that the degradation kinetics of volatile organic compounds supported in sonicated electrochemical tests are due to the increase of mass transport, minimization of the electrode fouling, and the combined generation of ^•^OH [40]. Here, we evaluated the sonolysis impact on degradation during sonolyisis assisted electro-Fenton of 4-CP in the means of: 1) H_2_O_2_ and ^•^OH formation, 2) changes in temperature, and 3) possible contribution of micromixing [7,28,35]. During EF, US and SEF, we tested the temperature changes, DO, H_2_O_2_ and ^•^OH (Table 3).

##### US impact on reactive species formation.

The negligible change in 4-CP concentration during sonolysis indicates that under tested conditions there is no cavitation bubble impact (pyrolysis and radical formation), and that the temperature increase (+8.7°C) has minimal impact on 4-CP volatilization. The H_2_O_2_ production was limited (up to 0.3 mg/L at 180 min comparing to 9.9 mg/L during EF) with negligible ^•^OH formation (confirmed by BA decrease and pHBA formation). Previous studies show that sonolytic systems with a higher frequency than that used in this study have proven to be effective in improving organic compound removal efficiency [1,40,51].

The application of US along with electrochemical process slightly increased formation of H_2_O_2_ to 1 mg/L at 180 min when Pd catalyst was absent compared to 0.3 mg/L with only sonolysis. When the Pd catalyst was applied, the H_2_O_2_ formation reached 9.8 mg/L 180 min following the kinetics of EF (b), after which it declined rapidly to 7.8 mg/L. It was also found that the production of ^•^OH based on reaction with BA was negligible when solely applied through sonolysis and coupled with electrochemical processes. Although studies have shown that US supports creation of H_2_O_2_ especially in presence of O_2_ and that O_2_ can conserve ^•^OH radicals to generate other oxidizing species like ^•^O and OH2 [35,36], the results under the conditions tested here are in an agreement with other studies showing negative effect on H_2_O_2_ formation. For example, Ziembowicz et al. found that initial H_2_O_2_ plays a crucial role in the effectiveness of the combined process (similar to the combined process used here) [52]. It was found that when initial concentration of H_2_O_2_ increases above 0.1 mM (compared to 0.3 mM formed under tested conditions here), the amount of H_2_O_2_ formed under US decreases significantly. The results given in Table 3 indicate that enhancement of EF by sonolysis relies on processes other than enhancement of H_2_O_2_ and ^•^OH formation, and relies on loss due to volatilization during temperature increase.

##### Impact of temperature changes.

During SEF tests, we monitored the temperature changes and evaluated the impact of temperature on the 4-CP removal since we applied pulsed sonification in order to increase practicality of the applied coupled treatment and avoid the use of a cooling bath [53–55]. Although temperature increase had a negligible impact of 4-CP degradation/volatilization sonolysis, temperature increase has been found to have an impact on rates of reactions via Fenton process [38]. The changes in the solution temperature over time with different amplitudes and ON/OFF ratios indicate a temperature increase from 2.5°C to 8.7°C with increasing the ultrasonic amplitudes from 10% to 70% under ON/OFF=0.1, and 3.5°C to 7°C from 10% to 70% with increasing the ultrasonic amplitudes from 10% to 30% under ON/OFF=0.2. The temperature increase under EF was within 2°C. These results imply that temperature changes during SEF had negligible impact on 4-CP removal since similar temperature variations occurred under amplitude of 70% with ON/OFF=0.1 and 30% with ON/OFF=0.2 while removal efficiency and kinetics significantly differ: >99.9% and 87.0%, respectively.

##### US contribution to mass transfer.

The results indicate that there is no impact of US on overall 4-CP removal in SEF due to increases in reactive species production and temperature changes. This was expected, because higher frequencies are responsible for reactive species formation and pulses allowed the temperature control. Monnier et al. found that micromixing through acoustic cavitation was more important at 20 kHz than at higher frequencies (540 kHz or 1 MHz) [7,56], while acoustic streaming has more impact at higher frequencies [57]. Besides temperature increase and chemical effects of violent bubble collapse, studies have shown that the collapse in the electrolyte-containing particles larger than 150 µm during US can produce shock waves and micro-jets, which cause powders to act as sonochemical catalysts through chemical, physical or combined mechanisms [26,30,40]. Micromixing causes significant increase of the mass transport (accelerating undissolved solute and impurity particles to several hundred meters per second), which enhances the rates of reactions [7,26,28,30,40,49,56]. Due to the presence of Pd powder and applied frequency of US (20kHz), it is reasonable to suggest that the micro-jetting is the primary cause of the enhancement of 4-CP removal under SEF compared to EF tested in this study. Our results indicate that the production of H_2_O_2_ (primary Pd catalyzed reaction) was not enhanced by US application although impacts of micro-jets include the dispersion of the catalysts and increase of the reactive area [7,28,58]. The improved fluid mixing by applied US is found to impact the rate of different chemical reactions even under mixing (as applied here) [11,25,51], and is suggested as a main impact of US on the SEF removal of 4-CP under tested conditions in this study. For example, Jordens et al. found that the best micromixing was with a 24 kHz probe and high power intensities [57] which is in agreement with 4-CP profile (Figure 7). Further investigations are needed to confirm the micromixing impacts on SEF and evaluate the parameters to enhance this effect on contaminant removal.

## 4. CONCLUSION

In this study, the performance of EF, US and SEF processes on 4-CP degradation were compared. Fe^2+^ concentration, Pd/Al_2_O_3_ catalysts concentration, initial pH and current density, and background electrolyte were evaluated in batch EF system. Optimum values were selected and SEF tests were performed with a 20KHz US instrument wave amplitude. Sonolysis of the contaminant was performed under optimum amplitudes. Optimum operating conditions for EF process are 80 mg L^−1^ Fe^2+^, 10 mg L^−1^ Pd/Al_2_O_3_, initial pH 3, 10 mM Na_2_SO_4_ and 16.94 mA cm^−2^ current density. Under these conditions, removal of 4-CP within 180 minutes was highest by SEF (>99.9%), followed by EF (83%) and US only (1.85%). Since radical formation was found to be absent under the conditions tested (20kHz), the sonolysis impacted the 4-CP removal probably due to increasing the mass transfer. The US increased the temperature by 8.7°C which did not impact the 4-CP volatilization or degradation. These results show that low level US frequency through pulses could be used to support electro-Fenton reaction by improving the reaction rates without the need for the electrolyte cooling.

## Figures and Tables

**Table 1.** EF test experiments design

Fe^2+^ Conc. (mg L^−1^)	Pd/Al_2_O_3_ (mgL^−1^)	Initial pH	Current density (mA cm^−2^)
0	10	3	16.94
19	10	3	16.94
40	10	3	16.94
80	10	3	16.94
80	10	3	Control
80	10	3	5.08
80	10	3	10.16
80	10	3	16.94
80	0	3	16.94
80	5	3	16.94
80	10	3	16.94
80	10	3	16.94
80	10	4	16.94
80	10	5	16.94

**Table 2.** Batch tests results

Parameter	Removal %	Zero-order decay rate constant min^−1^	R^2^
0 ppm Fe^+2^	11%	0.0004	0.99
19 ppm Fe^+2^	65%	0.0028	0.99
40 ppm Fe^+2^	79%	0.0031	0.97
80 ppm Fe^+2^	>99.9%	0.0043	0.98
Current density = Control	3.23%	0.0001	0.33
Current density = 5.08 mA cm^−2^	44%	0.0021	0.98
Current density = 10.16 mA cm^−2^	77%	0.0034	0.98
Current = 16.94 mA cm^−2^	>99.9%	0.0043	0.98
Pd/Al_2_O_3_ = 0 mg/l	24%	0.0012	0.96
Pd/Al_2_O_3_ = 5 mg/l	64%	0.0028	0.99
Pd/Al_2_O_3_ = 10 mg/l	>99.9%	0.0043	0.98
pH=3	>99.9%	0.0043	0.98
pH=4	86%	0.0035	0.98
pH=5	62%	0.0027	0.99
10 mM Na_2_SO_4_	>99.9%	0.0043	0.98
10 mM NaHCO_3_	65%	0.0029	0.99
10 mM NaNO_3_	67%	0.0025	0.95

**Table 3.** The changes in temperature, DO, H_2_O_2_ and ^•^OH formation during EF, US and SEF (at 180 min)

Treatment	H_2_O_2_ (mg/L)*	DO (change compared to initial) (mg/L)	pHBA/BA (%)**	BA decay (%)**	T (change compared to initial) (°C)
EF	9.9	+2.44	8.8	38.6	+1.9
US	0.3	−0.99	3.7	4.2	+10.0
SEF	7.8	+1.88	9.9	35.6	+11.4

* No Fe(II) added

** 80 mg/L Fe(II) added
